# Socioeconomic inequality and obesity prevalence trends in luxembourg, 1995–2007

**DOI:** 10.1186/1756-0500-5-467

**Published:** 2012-08-29

**Authors:** Anastase Tchicaya, Nathalie Lorentz

**Affiliations:** 1 Population and Employment Department, Centre d’études de populations, de pauvreté et de politiques socioéconomiques/International Networks for Studies in Technology, Environment, Alternatives, Development (CEPS/INSTEAD), Fonte Avenue 3, Esch/Alzette, L-4364, Luxembourg

**Keywords:** Obesity, Body Mass Index, Socioeconomic inequalities, Luxembourg

## Abstract

**Background:**

Overweight and obesity are becoming increasingly critical problems in most developed countries. Approximately 20% of adults in most European countries are obese. This study examines the prevalence of overweight and obesity in Luxembourg and their association with different demographic, socioeconomic (SES), and behavioural factors.

**Methods:**

The data used in this study were taken from 2 surveys on household income and living conditions conducted in 1995 and 2007. The target population was household residents aged 16 years and older, and body mass index (BMI) data were self-reported. Average BMI, overweight, and obesity prevalence rates were calculated according to each demographic (gender, nationality, marital status), SES (educational level, profession, and place of residence), and behavioural (physical activity and diet) factors. A multivariate logistic regression analysis was conducted to measure the relationship between obesity and demographic, SES, and behavioural factors. All analyses were conducted according to gender, and data used were weighted.

**Results:**

Between 1995 and 2007, the average BMI remained nearly constant among men and women in the entire study population. Obesity prevalence increased by 24.5% through the study period (14.3% in 1995 to 17.8% in 2007). Obesity prevalence increased by 18.5% for men (15.1% in 1995 to 17.9% in 2007) and by 30% for women (13.6% in 1995 to 17.7% in 2007). Between 1995 and 2007, obesity increased sharply by 48.2% (from 11% to 16.3%) in Portuguese men, 76.7% (from 13.3% to 23.5%) in Portuguese women, 79.7% (from 17.2% to 30.9%) in widowed men, and 84.3% (from 12.1% to 22.3%) in divorced women. Multivariate logistic regression analysis showed that the relationship between the educational level and obesity was not statistically significant for men, but was significant for women.

**Conclusions:**

The prevalence of overweight and obesity is high in Luxembourg and has changed slightly in recent years. SES inequalities in obesity exist and are most compelling among women. The fight against obesity should focus on education, with emphasis on the socially disadvantaged segment of the population.

## Background

Overweight and obesity are gradually becoming highly critical health problems in most developed countries [1]. In 1997, the World Health Organization (WHO) reported that obesity is a chronic disease and that ‘it is now so widespread that it replaces the traditional public health problems such as malnutrition and infectious diseases, and is one of most important factors of ill health’. In 2006, the Ministerial Conference of WHO found that obesity prevalence had tripled in 20 years; 1 of 2 adults and 1 of 5 children were overweight in the WHO European Region [2], and approximately 20% of adults in most European countries were obese [3]. The potential medical and socioeconomic (SES) consequences of overweight and obesity threaten individual health and health systems [4-7]. Several studies have examined the relationship between demographic and SES factors and obesity [8-14]. Among the SES factors, educational level plays an important role [8-14]. Most studies show a generally linear relationship (negative) between educational level and obesity, with the most educated people having low rates of obesity [9-14].

The relationship between SES and obesity is also mediated by behavioural factors such as diet (or nutrition) and physical inactivity, which play a role in the SES-body mass index (BMI) gradient [15-18]. However, in a population-based study in Sweden, only a part (18%–29%) of the association between educational level and obesity could be explained by the measured lifestyle factors [13].

In underprivileged zones and among the most disadvantaged groups, individuals (especially young people) are more inclined to smoke, which increases risk for overweight [19-21].

In Luxembourg, the prevalence of overweight and obesity was 36% and 17%, respectively, in 2005 [22]. A recent study [23] involving patients who underwent coronary angiography in 2008 and 2009 showed high rates of overweight (47.1%) and obesity (34.2%). Because the prevalence of these conditions is increasing in most developed countries, we studied their evolution and determinants between 1995 and 2007 in the general population in Luxembourg.

This paper has 2 main objectives. The first is to provide an account of the extent of overweight and obesity and their evolution in Luxembourg between 1995 and 2007, according to demographic and SES characteristic. The second is to analyse the relationship between demographic and socioeconomic determinants, behavioural factors, and obesity. This is the first study in Luxembourg to focus on the evolution of overweight and obesity in the general population. Owing to the diversity in the resident population (approximately 43.2% of residents were foreigners in 2007 according to the National Institute of Statistics and Economic Studies [24]), we were able to determine differences in the prevalence of overweight and obesity between Luxembourg natives and foreigners.

## Methods

The data analysed in this study pertain to household income and living conditions from ‘Panel Socio-Economique Liewen zu Lëtzebuerg 2/European Community Household Panel’ and ‘Panel Socio-Economique Liewen zu Lëtzebuerg 3/European Union-Statistics on Income and Living Conditions’ surveys conducted in 1995 and 2007. This type of longitudinal survey concerns only private households; the survey technique is based on face-to-face interviews, and the target population is people aged 16 years and older. The data represent 5,117 people in 1995 and 7,768 people in 2007; the sample population comprised 51.7% women in 1995 and 50.8% women in 2007. We used cross-sectional weighted data for the analyses.

### Data

BMI, age, gender, marital status, nationality, education level, profession, place of residence, physical activity, and diet were studied (Table 1, Table 2). BMI, defined as the ratio between a person’s weight and height (kg/m^2^), was self-reported. To minimize bias, the investigator handed the respondents a card that showed the corresponding BMI value at the intersection of their height and weight. Overweight was defined as a BMI of 25–30 kg/m^2^, and obesity was defined as a BMI of ≥ 30 kg/m^2^.

**Table 1:** Prevalence of obesity for the men in 1995 and 2007, by demographic, socio-economic and behavioural factors

		**1995**				**2007**			
		**N**	**Average BMI (SD)**	**Overweight**	**Obese**	**N**	**Average BMI (SD)**	**Overweight**	**Obese**
**Total**		5117	25.1 (4.4)	37.3	14.3	7768	25.5 (4.6)	36.4	17.8
**Men**									
**All**		2471	25.9 (4.0)	46.3	15.1	3821	26.0 (4.1)	43.9	17.9
**Age groups**	16-24	178	24.0 (2.8)	34.7	4.9	519	23.4 (3.9)	26.2	7.0
	25-34	585	24.8 (3.5)	38.8	9.7	651	25.1 (3.3)	42.8	11.4
	35-44	560	26.0 (4.0)	47.1	15.9	827	25.9 (3.5)	46.7	15.4
	45-54	431	26.7 (4.4)	52.7	18.6	748	26.9 (4.5)	47.0	22.1
	55-64	344	27.0 (3.9)	52.8	20.9	497	27.4 (4.1)	45.3	29.1
	65+	374	26.2 (4.1)	49.3	17.6	579	27.0 (4.5)	51.5	23.4
**Nationality**	Luxembourgers	1827	25.9 (4.1)	45.1	15.8	2328	26.1 (4.8)	43.9	18.7
	Portuguese	194	25.3 (3.0)	46.4	11.0	561	25.7 (3.4)	44.2	16.3
	French	94	26.3 (4.4)	51.8	13.8	188	24.9 (2.7)	37.5	10.5
	German	41	26.4 (3.5)	58.6	17.2	81	26.5 (3.0)	52.4	17.1
	Others	315	25.7 (3.5)	50.1	13.6	664	25.9 (3.6)	44.0	18.5
**Marital status**	Single	646	24.7 (3.4)	36.6	9.5	1184	24.3 (3.9)	33.4	8.8
	Married	1623	26.3 (4.1)	49.7	17.2	2294	26.7 (3.9)	49.3	21.7
	Divorced/separated	100	26.2 (4.2)	53.8	13.9	239	26.3 (3.4)	40.2	20.5
	Widowed	102	26.0 (4.4)	47.6	17.2	104	28.4 (5.5)	50.7	30.9
**Educational level**	Primary	882	26.2 (4.1)	45.8	18.6	900	26.3 (3.9)	46.1	20.3
	Secondary	1054	25.7 (3.8)	46.6	13.4	2015	26.1 (4.5)	44.4	18.5
	Tertiary	523	25.5 (3.9)	46.4	12.3	869	25.2 (3.4)	39.9	12.8
**Profession**	Managers and Intellectual professions	479	25.5 (4.0)	47.4	12.1	642	25.2 (3.2)	46.5	9.0
	Intermediate Professions	339	25.7 (3.7)	48.3	14.0	519	26.2 (4.4)	45.7	18.8
	Employed	169	25.4 (3.5)	40.8	12.6	388	25.9 (3.8)	46.0	15.3
	Agricultural workers/Craftsmen/ Workers/No profession	1483	26.1 (4.0)	46.2	16.6	2271	26.2 (4.3)	42.3	20.6
**Place of residence**	Centre- North	271	25.8 (4.3)	48.4	11.2	474	26.2 (4.8)	35.7	21.3
	Centre- South	1238	26.0 (4.0)	46.1	15.9	1841	25.8 (3.9)	47.2	15.9
	East	230	25.9 (4.1)	48.5	13.8	438	26.4 (4.6)	43.1	17.3
	North	72	27.0 (3.9)	46.9	26.4	235	26.4 (4.3)	43.3	25.4
	West	185	25.9 (4.0)	44.3	17.8	241	27.3 (3.8)	49.7	27.0
	Luxembourg-city	425	25.3 (3.5)	45.1	12.8	592	25.3 (3.8)	38.3	15.0
**Physical activity**	Yes	991	25.4 (3.7)	47.2	10.7	2041	25.3 (3.8)	44.1	12.6
	No	1471	26.2 (4.0)	46.0	18.1	1779	26.7 (4.3)	43.5	23.9
**Diet**	Balanced	2040	25.7 (3.9)	47.0	13.9	3265	25.8 (4.1)	43.8	17.0
	Unbalanced	308	26.7 (4.5)	44.5	23.1	471	27.1 (4.3)	43.9	24.5
	No know	118	25.8 (3.7)	40.5	14.8	81	26.1 (3.7)	49.0	17.2

**Table 2:** Prevalence of obesity for the women in 1995 and 2007, by demographic, socio-economic and behavioural factors

		**1995**				**2007**			
		**N**	**Average BMI (SD)**	**Overweight**	**Obese**	**N**	**Average BMI (SD)**	**Overweight**	**Obese**
**Total**		5117	25.1 (4.4)	37.3	14.3	7768	25.5 (4.6)	36.4	17.8
**Women**									
**All**		2649	24.4 (4.6)	28.9	13.6	3947	25.1 (5.0)	29.3	17.7
**Age groups**	16-24	180	23.0 (4.2)	15.7	9.8	469	22.0 (3.4)	15.4	4.8
	25-34	582	23.1 (4.6)	19.7	10.1	678	23.7 (3.8)	22.8	11.2
	35-44	527	23.9 (5.0)	23.7	12.7	859	24.9 (5.2)	23.4	16.1
	45-54	404	25.0 (4.2)	32.8	14.7	717	25.8 (5.2)	29.9	21.9
	55-64	362	26.0 (4.0)	40.9	19.7	471	26.3 (4.6)	38.1	21.7
	65+	592	25.1 (4.8)	36.6	14.6	754	27.2 (5.8)	44.4	27.0
**Nationality**	Luxembourgers	1912	24.4 (4.7)	29.6	13.9	2462	25.2 (5.7)	30.2	18.5
	Portuguese	223	24.7 (4.1)	29.9	13.3	462	25.9 (4.1)	27.3	23.5
	French	101	23.2 (3.7)	31.5	5.6	191	22.8 (2.7)	20.1	6.4
	German	51	24.3 (5.1)	30.3	15.2	105	25.0 (4.5)	21.5	18.7
	Others	359	24.3 (4.6)	23.8	14.2	726	24.8 (4.4)	31.0	14.1
**Marital status**	Single	565	23.2 (4.8)	18.7	11.5	948	23.1 (4.4)	19.8	9.5
	Married	1457	24.5 (4.4)	31.5	12.7	2271	25.5 (4.8)	30.9	19.0
	Divorced/separated	171	24.2 (4.8)	28.0	12.1	325	25.7 (5.1)	27.5	22.3
	Widowed	454	25.6 (4.8)	33.8	19.7	403	27.1 (5.7)	43.8	26.1
**Educational level**	Primary	1491	25.3 (4.6)	33.5	18.6	1235	26.8 (5.4)	36.6	25.2
	Secondary	866	23.5 (4.4)	23.9	8.6	1901	24.8 (5.0)	27.2	16.9
	Tertiary	284	22.2 (4.2)	20.0	2.8	788	23.4 (3.6)	23.5	7.7
**Profession**	Managers and Intellectual professions	175	22.7 (4.6)	26.4	3.3	382	24.2 (4.1)	30.0	10.8
	Intermediate Professions	207	22.7 (5.0)	18.5	6.8	462	23.6 (4.7)	18.2	11.4
	Employed	268	23.0 (4.4)	14.0	9.6	500	23.9 (4.3)	26.9	10.2
	Agricultural workers/Craftsmen/Workers/No profession	1997	24.9 (4.5)	32.2	15.8	2603	25.8 (5.2)	31.6	21.3
**Place of residence**	Centre- North	295	24.1 (5.2)	27.3	13.0	461	25.0 (5.4)	28.5	14.7
	Centre- South	1277	24.4 (4.4)	30.1	13.3	1933	25.3 (4.9)	29.1	19.0
	East	285	24.9 (5.1)	25.4	19.5	402	25.4 (4.7)	29.5	20.2
	North	73	24.2 (3.8)	30.3	10.8	225	25.1 (5.1)	33.4	15.9
	West	198	24.7 (4.9)	28.7	15.7	249	25.4 (5.4)	24.3	22.3
	Luxembourg-city	518	24.1 (4.6)	28.6	11.1	677	24.5 (4.7)	30.5	13.5
**Physical activity**	Yes	782	23.2 (4.2)	21.0	9.5	1973	24.0 (4.5)	25.1	11.6
	No	1847	24.9 (4.7)	32.0	15.3	1974	26.2 (5.1)	33.5	23.8
**Diet**	Balanced	2264	24.3 (4.4)	29.3	12.4	3479	25.1 (4.9)	29.6	17.3
	Unbalanced	300	25.0 (5.6)	28.6	19.8	403	25.6 (5.5)	26.9	21.7
	No know	80	25.4 (6.1)	18.9	26.7	57	25.3 (4.5)	26.3	19.3

Marital status was identified by 4 modalities: single, married, separated/divorced, and widowed. The nationality variable represented national origin of subjects residing in Luxembourg. The International Standard Classification of Education (ISCED) was used to describe the educational level, which was divided into 3 categories: primary education (ISCED 1), secondary education (ISCED 2 and ISCED 3), and tertiary education (ISCED 4 and ISCED 5).

Profession was defined using the international classification of the International Labour Organization, which is based on the last occupation of the respondents and grouped into 4 terms: managers and intellectual professions, intermediate professions, employed, and agricultural workers/craftsmen/workers/no profession.

The residence location variable allowed researchers to measure spatial variation in the prevalence of overweight and obesity: this variable comprised the following 6 regions: Centre-North, Centre-South, East, West, North, and Luxembourg City.

Physical activity was defined as a dichotomous variable: Respondents answered yes if physical activity was practiced and no if not. Diet was measured by the question: ‘Do you think your diet is balanced, unbalanced, or you do not know’?

### Statistical Analysis

The prevalence of overweight and obesity and average BMI were calculated according to demographic (gender, nationality, and marital status) SES (educational level, profession, and residence location), and behavioural (physical activity and diet) factors (Table 1, Table 2). Multivariate logistic regression analysis was conducted to measure the relationship between obesity and demographic SES and behavioural factors (Table 3). Obesity was the interest variable and the independent variables were age, nationality, marital status, educational level, profession, place of residence, physical activity, and diet. All analyses were conducted separately by year and gender, and data used were weighted. All analyses were performed using the SAS^©^ System (SAS Institute, Cary, N.C., USA).

**Table 3:** Association of the obesity with the demographic, socio-economic and behavioural factors, by gender, in 1995 and 2007

		**1995**				**2007**			
		**Men OR [CI]**		**Women OR [CI]**		**Men OR [CI]**		**Women OR [CI]**	
**Age groups**	16-24	1 (ref.)		1 (ref.)		1 (ref.)		1 (ref.)	
	25-34	1.99	[0.93-4.27]	1.21	[0.67-2.20]	1.36	[0.85-2.18]	3.38	[1.96-5.82]*
	35-44	3.14	[1.44-6.86]*	1.49	[0.80-2.79]	1.46	[0.90-2.38]	4.64	[2.72-7.93]*
	45-54	3.88	[1.75-8.60]*	1.60	[0.83-3.08]	2.26	[1.39-3.69]*	6.82	[3.99-11.64]*
	55-64	4.02	[1.79-9.04]*	1.90	[0.97-3.73]	3.08	[1.86-5.09]*	6.01	[3.45-10.48]*
	65+	2.82	[1.24-6.43]*	0.96	[0.48-1.90]	1.86	[1.11-3.12]*	6.57	[3.78-11.40]*
**Marital status**	Married	1 (ref.)		1 (ref.)		1 (ref.)		1 (ref.)	
	Single	0.76	[0.53-1.08]	1.31	[0.90-1.91]	0.47	[0.34-0.64]*	1.11	[0.82-1.50]
	Divorced/separated	0.69	[0.38-1.26]	0.88	[0.53-1.47]	0.81	[0.56-1.16]	1.28	[0.95-1.72]
	Widowed	0.90	[0.51-1.58]	1.57	[1.12-2.21]*	1.59	[0.99-2.53]	0.97	[0.73-1.30]
**Nationality**	Luxembourgers	1 (ref.)		1 (ref.)		1 (ref.)		1 (ref.)	
	French	0.77	[0.41-1.48]	0.46	[0.19-1.12]	0.71	[0.43-1.19]	0.43	[0.23-0.79]*
	German	1.30	[0.56-3.01]	1.71	[0.76-3.88]	1.23	[0.66-2.32]	1.30	[0.76-2.20]
	Others	0.87	[0.60-1.26]	1.20	[0.84-1.71]	1.10	[0.85-1.41]	0.86	[0.67-1.11]
	Portuguese	0.62	[0.36-1.06]	0.90	[0.56-1.43]	0.82	[0.60-1.12]	1.30	[0.97-1.75]
**Educational level**	Tertiary	1 (ref.)		1 (ref.)		1 (ref.)		1 (ref.)	
	Primary	1.45	[0.99-2.13]	5.03	[2.29-1.07]*	0.76	[0.54-1.06]	2.06	[1.43-2.98]*
	Secondary	1.02	[0.71-1.47]	2.36	[1.07-5.20]*	0.86	[0.65-1.14]	2.08	[1.48-2.92]*
**Profession**	Managers and intellectual Professions	1 (ref.)		1 (ref.)		1 (ref.)		1 (ref.)	
	Agricultural workers/Craftsmen/Workers/No profession	1.36	[0.91-2.02]	2.61	[1.06-6.47]*	2.79	[1.93-4.02]*	1.19	[0.79-1.81]
	Employed	1.07	[0.58-1.96]	2.03	[0.76-5.46]	2.44	[1.58-3.79]*	0.55	[0.34-0.90]*
	Intermediate Professions	1.20	[0.76-1.88]	1.66	[0.59-4.65]	2.64	[1.79-3.90]*	0.80	[0.50-1.29]
**Place of residence**	Luxembourg-city	1 (ref.)		1 (ref.)		1 (ref.)		1 (ref.)	
	Centre- North	0.84	[0.51-1.37]	1.23	[0.78-1.95]	1.51	[1.07-2.13]*	1.10	[0.77-1.58]
	Centre- South	1.09	[0.77-1.53]	1.09	[0.78-1.52]	0.99	[0.75-1.30]	1.35	[1.04-1.76]*
	East	0.94	[0.59-1.50]	1.93	[1.26-2.94]*	1.03	[0.72-1.47]	1.50	[1.05-2.13]*
	North	2.27	[1.21-4.24]*	0.78	[0.35-1.76]	1.97	[1.31-2.95]*	1.20	[0.77-1.87]
	West	1.15	[0.69-1.91]	1.13	[0.68-1.88]	1.84	[1.24-2.72]*	1.73	[1.16-2.58]*
**Physical activity**	Yes	1 (ref.)		1 (ref.)		1 (ref.)			1 (ref.)
	No	1.54	[1.19-1.98]*	1.18	[0.88-1.58]	1.80	[1.49-2.18]*	1.78	[1.48-2.15]*
**Diet**	Balanced	1 (ref.)		1 (ref.)		1 (ref.)		1 (ref.)	
	Unbalanced	2.19	[1.60-3.00]*	2.06	[1.48-2.87]*	1.99	[1.54-2.57]*	1.62	[1.23-2.15]*
	No know	0.95	[0.54-1.67]	2.47	[1.43-4.25]*	1.35	[0.73-2.51]	0.94	[0.47-1.91]
***R***^***2 ***^***adjusted***		***R***^2 ^***0.0821***	***R***^2 ^***0.1060***	***R***^2 ^***0.1341***	***R***^2 ^***0.1328***				

Results are expressed as Odds Ratio (OR) and 95% Confidence Interval (CI). Statistical analysis was performed by multivariate logistic regression.

(*) indicated that OR were statistically significant at 95% CI.

### Ethical consideration

This research was conducted in accordance with the World Medical Association Declaration of Helsinki. The Luxembourg National Commission for Data Protection approved the EU Survey on Income and Living Conditions, which also was used.

## Results

### Distribution of BMI and prevalence of overweight and obesity, stratified by year and gender (Table 1, Table 2)

BMI data were stable between 1995 and 2007 for both men and women in the entire study population. It was 26.0 kg/m^2^ for men in 2007 (25.9 kg/m^2^ in 1995) and 25.1 kg/m^2^ for women in 2007 (24.4 kg/m^2^ in 1995). Men had a mean BMI of 23.4 to 27.4 kg/m^2^ in 2007 and were overweight between the ages of 25 and 34, on an average. For women, mean BMI ranged between 22.0 and 27.2 kg/m^2^ and women aged 45 and older, on average, were overweight.

Weight distribution varied by gender and age: the percentage of overweight men decreased from 46.3% in 1995 to 43.9% in 2007 and obesity in men increased from 15.1% in 1995 to 17.9% in 2007 (an 18.5% increase in 12 years). Overweight prevalence ranged from 26.2% to 51.5% in 2007 among younger and older men, while obesity prevalence ranged from 7.0% to 29.1% in 2007, respectively, among men aged 16 to 24 and 55 to 64 years. Obesity prevalence was highest among older men (+8 percentage points).

Among women, the prevalence of overweight increased from approximately 28.9% in 1995 to 29.3% in 2007 and increased with age, ranging between 15.4% and 44.4% in 2007. Obesity prevalence increased to approximately 17.7% in 2007 from 13.6% in 1995 and varied between 4.8% and 27% in 2007. The prevalence of obesity among women increased to 30% between 1995 and 2007.

Between 1995 and 2007, the prevalence of overweight remained stable among both men and women, while the prevalence of obesity increased, particularly among women age 65 years and older (an 85% increase) and among those aged 45 to 54 years (a 49% increase). Obesity prevalence increased by 42.9% (4.9% in 1995 to 7.0% in 2007) in men aged 16–24 years, while it decreased by 51% (9.8% in 1995 to 4.8% in 2007) among women of the same age group between 1995 and 2007.

In 2007, Luxembourg men had a higher obesity prevalence rate than the mean value, followed by Portuguese men. Among women, the prevalence rate was higher than the mean in Portuguese women. Between 1995 and 2007, the highest increase in obesity prevalence was observed in Portuguese women (from 13.3% to 23.5%, or a 76.7% increase) and Portuguese men (from 11.0% to 16.3%, or a 48.2% increase).

Obesity prevalence was highest among widowed men (30.9%) and widowed women (26.1%) in 2007. Compared to 1995, obesity prevalence increased to 79.7% in men and 32.5% in women in 2007. Regarding educational level, higher obesity prevalence was observed in those who completed their primary education compared to those who completed their graduation. Obesity prevalence ratios were highest among women (3.27 for women and 1.59 for men). Large increases in obesity prevalence were found among women. In 2007, obesity prevalence among agricultural workers, farmers, craftsmen, and other workers was approximately twice that observed among managers working in intellectual professions for both men and women. There was generally an increased prevalence of obesity in each profession between 1995 and 2007, except for male managers. Moreover, obesity prevalence increased according to the location of residence between 1995 and 2007, with the exception being men living in the North region. The increase in obesity prevalence was higher among those who did not engage in physical activity between 1995 and 2007 (32% for men [from 18.1% in 1995 to 23.9% in 2007] and 55.6% for women [from 15.3% in 1995 to 23.8% in 2007]).

Men and women who say their diet is balanced are less likely to be obese than people who do not believe they eat a balanced diet. Obesity prevalence was higher among those who rated their diet as unbalanced (22.3% in men [from 23.1% in 1995 to 24.5% in 2007] and 39.5% for women [from 19.5% in 1995 to 21.7% in 2007]).

### Link between demographic and SES determinants and obesity (Table 3)

Risk for obesity was associated with age among both men and women in 2007. This association was absent in women in 1995 and stronger in women than in men in 2007, as shown by the odds ratio (OR).

Marital status was not associated with risk for obesity in both men and women in 1995 and 2007. However, married men were more likely to be obese than single men in 2007, and widowed women were more likely to be obese than married women in 2005.

Nationality was not associated with obesity prevalence among men in 1995 and 2007 or among women in 1995. A statistically significant difference appeared between French women and Luxembourg women in 2007; the French women were less likely to be obese than the Luxembourg women (OR = 0.43, confidence interval [CI] = 0.23–0.79).

Educational level was associated with the likelihood of obesity among women in both 1995 and 2007. Women with an education level below the tertiary education were twice as likely to be obese in 2007 (OR = 2.06, CI = 1.43–2.98 for primary education, and OR = 2.08, CI = 1.48–2.92 for secondary education). Between 1995 and 2007, the strength of the association between education and obesity decreased among women; however, the extent of inequality remained high.

The association between profession and obesity was statistically significant among men in 2007 and among women in 1995.

The location of residence was associated with obesity prevalence for both men and women. Compared to residents of Luxembourg City, men who inhabited the regions of Centre North, North, and West generally were at an approximately twofold higher risk for being obese, and women living in the Centre South, East, and West regions had a less-than-twofold risk.

Lack of physical activity was associated with the likelihood of being obese in 2007 for both men and women. Men and women who did not exercise were nearly 1.8 times more likely to be obese than those who exercised. In 1995, this link was only statistically significant in men.

Links between diet and obesity were statistically different among men and women in 1995 and 2007. People who were considered to have unbalanced nutrition were more likely to be obese than those with a balanced diet.

## Discussion

Our results show that obesity prevalence increased by 24.5% through the study period in Luxembourg. However, because the prevalence may seem stable overall, increases across different ages may be overlooked. Age remained an obesity inequality factor for men and women in 2007, but only for men in 1995. Women tended to pay more attention to their weight than men did; therefore, less than 50% of the women were overweight between 1995 and 2007. These trends were observed in other developed countries as well. In France, the prevalence of adult obesity increased by 52.3% between 1997 and 2006 (13.1% in 2006 and 8.6% in 1997) [25]. In Switzerland, 2 studies found that the prevalence rates of obesity were 14.1% in men and 16% in women in 2005 and 2006 [26], and 8.6% in men and 7.7% in women in 2007 [27]. In Greece, overall obesity prevalence was 22.3% (25.8% in men, 18.4% in women) in 2003 [28], which was higher than that in Luxembourg. Our results showed that obesity prevalence rates observed in both men and women in Luxembourg were lower than those in both the United States and England. In the United States, the age-adjusted prevalence of obesity was 32.2% among men and 33.5% among women [29]. In England, 26.1% of adults aged 16 years and older were obese [30].

Resident nationality reveals changes in lifestyle and dietary habits associated with population migration. For example, obesity prevalence in Portugal was lower (14.1% of men and 16% of women in 2005 and 2006) [26] than among Portuguese residents in Luxembourg. In contrast, for French residents in Luxembourg, obesity prevalence was lower than the prevalence rates observed in France (12.5% of men and 13.6% of women) [25].

The extent of the relationship between SES factors and obesity prevalence examined with multiple logistic regression analysis confirmed the results of other studies [4,8-14,23,28,31-33].

The influence of education on obesity is significant in women because it clearly shows the social gradient; other studies have presented results showing an inverse relationship between education level and SES status [8,10,31]. The link between obesity and educational level is reflected in the multivariate logistic regression model. Educational level influences the ability to process information regarding healthy lifestyle and, more specifically, overweight and obesity [8,32]. According to Kenkel [32], education helps people choose healthy lifestyles by improving their knowledge of the relationship between health behaviours and health outcomes. In rural Appalachia, a study found that education has a significant influence on risk for obesity [4]. Cawley and colleagues [10] emphasized the role of information and education in the association between education and obesity. A low level of education was an obesity risk factor in our study, particularly among women. Women who had only reached the level of primary or secondary education were twice as likely to be obese compared to those who had achieved a higher level of education. In Greece, educational level was not associated with risk for obesity among men, but this relationship existed among women [28]. In Spain, a study conducted between 1987 and 1997 showed a predisposition to obesity that was much higher in people with a basic level of education, regardless of gender [11]. Overall, these results confirm the important role of education in the prevalence of overweight and obesity, as observed in other studies [4,9,10,28].

Some studies found that married people were more likely to be obese than unmarried people [28,33]. Our results showed that marital status was associated with obesity among women in 1995 and among men in 2007. In Greece, married men and married women were twice as likely to be obese than those who were not married [28].

Our results demonstrated a larger difference in obesity prevalence between agricultural workers, craftsmen, general workers, and those with no profession, and men working as managers and in intellectual professions. The results of our multivariate analysis showed that men working in lower professions were more than twice as likely to be obese than men working in top professions (e.g., managers and intellectual professions) in 2007. This result was similar to that found in England, where higher professional status was associated with lower risk for obesity [34]. Among women, this difference decreased through the study period but remained high. This trend also was observed in France between 1992 and 2003 [35].

Obesity distribution is associated with respondents’ residence location; this association was higher in 2007. These differences may be attributable to SES context, diet and physical activity, and environment (urban or rural). In Portugal, a cross-sectional study showed that people who lived in rural areas were at lower risk for obesity than those who lived in urban areas [36]. In contrast, our results suggest that men and women who live outside of the major town of Luxembourg (Luxembourg City) are more likely to be obese. The North and West areas are less populated and include rural areas, and obesity prevalence was higher among men and women in these 2 areas. It is noteworthy that even in a small country such as Luxembourg, spatial variations of obesity can be observed.

Many studies have found that physical activity is associated with obesity [37,38]. Our findings showed that physical activity was associated with obesity risk in both men and women, particularly in 2007.

Other studies found a statistically significant association between obesity and diet, but sometimes the results were mixed [39-41]. Our results suggest the relationship between obesity and diet was statistically significant in both men and women in 1995 and 2007. These results may be of interest for those involved in diet-related health promotion.

Limitations of this study were as follows: BMI was self-reported, and we did not have data on smoking, alcohol consumption, or calorie intake. These missing data are behavioural obesity determinants (lifestyle) that are required to be controlled for in an analysis of the relationship between obesity and SES determinants.

## Conclusions

The prevalence of overweight and obesity is relatively high in Luxembourg; however, it changed only slightly between 1995 and 2007. SES inequalities associated with obesity are important, especially inequality in education among women and professional inequality among men. The fight against obesity should focus on educating the population at large, with particular emphasis on socially disadvantaged people. Our findings confirm the influence of lifestyle factors on obesity risk. A sustained policy to fight obesity is justified by the risks associated with this health problem, both in terms of medical expenses and morbidity [42].

## Competing interests

The authors declare that they have no competing interests.

## Authors’ contributions

AT conducted the statistical analysis and wrote the manuscript. NL conducted the statistical analysis and wrote the manuscript. All authors read and approved the final manuscript.
